# Feasibility Study of Precise Balloon Catheter Tracking and Visualization with Fast Photoacoustic Microscopy

**DOI:** 10.3390/s20195585

**Published:** 2020-09-29

**Authors:** Jahae Kim, Thi Thao Mai, Jin Young Kim, Jung-Joon Min, Chulhong Kim, Changho Lee

**Affiliations:** 1 Department of Nuclear Medicine, Chonnam National University Medical School & hospital, 42 Jebong-ro, Dong-gu, Gwangju 61469, Korea; jhbt0607@hanmail.net; 2 Department of Artificial Intelligence Convergence, Chonnam National University, 77 Yongbong-ro, Buk-gu, Gwangju 61186, Korea; 196286@jnu.ac.kr; 3 Departments of Creative IT Engineering and Electrical Engineering, Pohang University of Science and Technology (POSTECH), 77 Cheongam-ro, Nam-gu, Pohang, Gyeongbuk-do 37673, Korea; ronsan@postech.ac.kr (J.Y.K.); chulhong@postech.edu (C.K.); 4 Department of Nuclear Medicine, Chonnam National University Medical School & Hwasun Hospital, 264, Seoyang-ro, Hwasun-eup, Hwasun-gun, Jeollanam-do 58128, Korea; jjmin@jnu.ac.kr

**Keywords:** balloon catheter, photoacoustic imaging, image guiding

## Abstract

Correct guiding of the catheter is a critical issue in almost all balloon catheter applications, including arterial stenosis expansion, coronary arterial diseases, and gastrointestinal tracking. To achieve safe and precise guiding of the balloon catheter, a novel imaging method with high-resolution, sufficient depth of penetration, and real-time display is required. Here, we present a new balloon catheter guiding method using fast photoacoustic microscopy (PAM) technique for precise balloon catheter tracking and visualization as a feasibility study. We implemented ex vivo and in vivo experiments with three different medium conditions of balloon catheter: no air, air, and water. Acquired cross-sectional, maximum amplitude projection (MAP), and volumetric 3D PAM images demonstrated its capability as a new imaging guiding tool for balloon catheter tracking and visualization.

## 1. Introduction

A balloon catheter is commonly used for opening the narrow or blocked area in the body. The balloon catheter technique has been mostly applied in the field of coronary artery diseases. In stenotic coronary vessels, the contracted balloon enters the affected coronary artery. The balloon is inflated to widen the artery, squashing plaques against the artery wall. It can improve myocardial blood flow in a localized stenotic lesion when the deflated balloon is removed [1]. Similarly, it has been applied to peripheral arterial disease [2], extracranial or intracranial cerebral vascular lesion [3], stricture of the gastrointestinal tract [4], eustachian tube dysfunction [5], and ureteropelvic junction obstruction [6]. Unlike its function as an expander of the narrowed area, the balloon catheter can also function as a blocker at the site of ruptured or torn vessels. If a large amount of bleeding occurs from the aorta, the inflation of resuscitative balloon catheters can lead to blocking blood leakage from the perforated site [7]. Such the balloon catheter procedure can increase blood flow to the heart and brain, thus preventing cardiovascular collapse [8]. Recently, given a less-invasiveness of catheterization, a new hybrid operation which is combining open surgery and vascular intervention has been used in many cases such as heart disease [9], liver injury [10], subclavian artery injury [11], and lower extremity vascular injury [12]. The hybrid operation leads to minimizing the extent, the duration, and the cost of the traditional operation. In the hybrid operation, the balloon catheter technique still works as the main tool for vasculature intervention.

Whether the purpose of a balloon catheter is to open a blockage or block a torn site, it is necessary to check that the balloon catheter enters the body and that the balloon is located at the correct place in the body. To confirm the balloon’s location, X-ray fluoroscopy with contrast dye is regarded as a gold standard. However, X-ray fluoroscopy devices cause substantial radiation exposure to patients and operators [13]. Furthermore, iodine-containing contrast is essential for fluoroscopy-guided angiography. It has been shown the contrast agent has adverse clinical consequences due to its high osmolality [14,15]. In addition, the high viscosity of the contrast agent takes more time to complete the interventional procedure [16]. Due to its relatively poor spatial resolution, X-ray fluoroscopy is limited to show the precise location of the catheter. To overcome these limitations of X-ray fluoroscopy, there have been experimental studies of balloon catheters using interactive MRI [17], carbon dioxide digital subtraction angiography (CO_2_-DSA), [18] or ultrasound imaging (USI) [19]. However, MRI is not a proper solution to use in a real-surgical environment due to its huge size and limitation in the use of metal surgical tools known to interfere with the magnetic field. In addition, it is relatively expensive. CO_2_-DSA needs to use additional agents to visualize vasculatures. Although USI is a better option to use in urgent and surgical conditions, it has difficulties showing the catheter clearly, due to its angle-dependent artifact. Furthermore, these techniques do not have high enough resolution to visualize images below 50 µm. This is a bottleneck of precise catheter guiding. In particular, although many operating sites use intraoperative imaging, such as fluoroscopy for vascular intervention parts in hybrid operation, high resolution and rapid follow-up imaging techniques are still required to track the catheter guiding precisely.

In recent years, photoacoustic imaging (PAI) has been spotlighted as a promising bio-imaging technology with the development of high-performance PAI laser source and rapid computing technology. This technique is originated from the photoacoustic (PA) effect discovered by Alexander Graham Bell in 1880. When a nano-second pulsed laser shines on the targeting sample, it undergoes absorption of laser energy and thermal-elastic expansion. As a result of this process, broad acoustic waves appear. These waves can be captured by common ultrasound transducers. Finally, two or three-dimensional images can be obtained via reconstruction algorithms. Thanks to less scattering of ultrasound in biological tissue, PAI can achieve deep tissue imaging while maintaining ultrasonic resolution [20,21,22,23,24]. Furthermore, depending on aiming applications, PAI can provide multiscale images from several nanometers to several centimeters selectively by controlling systemic specifications between optical and ultrasonic [25]. Normally, PA microscopy (PAM) can provide superior spatial resolution and sensitivity in relatively shallowed regions with focusing illuminated laser beam or detecting ultrasonic beam [26,27,28,29]. In contrast, PA tomography (PAT) enables to achieve deep tissue imaging with multi-arrayed transducers and broad laser illumination without any mechanical scanning [30,31,32]. Additionally, based on intrinsic absorption composites in the body, PAI can be used to visualize not only structural parameters including vasculature networks, location of melanoma, the structure of tendons, plaques and so on, but also functional parameters including blood velocity, oxygen saturated level, and metabolism ratios [33,34]. Using these advantages, PAI has been widely utilized to improve basic/preclinical studies and clinical translation in various areas, such as oncology, dermatology, ophthalmology, neurology and so on [35,36,37].

PAI is also utilized as a powerful tool for a wide range of image-guided applications. For minimally invasive biopsy of sentinel lymph nodes (SLNs), a handheld array probe-based PAI system has demonstrated its performance of visualizing the ability of needle insertion and guiding to SLNs dyed methylene blue (MB) and indocyanine green (ICG) [38,39]. A similar clinical PAI system has been successfully developed to detect the catheter with higher sensitivity than USI [40]. These approaches have suppressed the drawbacks of USI guiding by clear visualization of the metal part of the needle and the catheter. PAI is also used to effectively delineate cancerous regions for photothermal therapy monitoring and drug delivery [41,42]. By showing the margin of cancer, PAI can enhance the therapeutic effect. Moreover, thanks to its real-time displaying capability, relatively deep penetration depth, and high spatial resolution, PAI has shown the feasibility of surgical imaging-guided applications such as a precise needle guiding to a single vessel, incision of melanoma tumor, drug injection into tumor, spinal fusing surgery and so on [43,44,45,46,47].

In this study, we conceptually demonstrated the capability of PAI to track and visualize the correct location of the balloon catheter in the blood vessel under open surgery conditions. Especially, we identify the location of the catheter in the procedure of the endovascular intervention part constituting hybrid operation and the location of the balloon without any contrast agents. Using the fast-MEMS based PAM (f-MEMS-PAM) system, three-dimensional and cross-sectional PAM images were successfully obtained in real-time. In particular, by setting up three injection medium conditions (i.e., no air, air and water) in the balloon, the balloon catheter was selectively tracked and visualized.

## 2. Materials and Methods

### 2.1. Balloon Catheter

**Figure 1:**
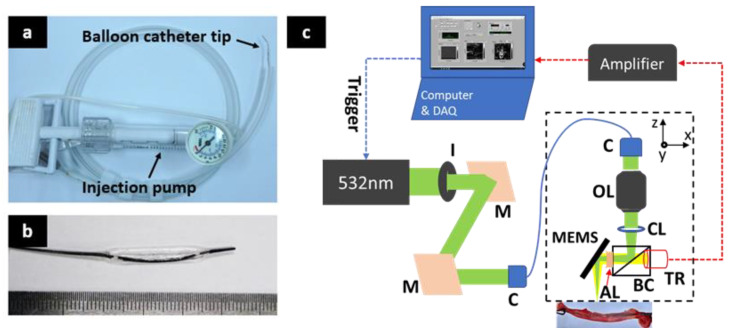
(**a**) Photography of the balloon catheter and the inflation pump, (**b**) Enlarged photograph of the tip of the balloon catheter, (**c**) Schematic of the fast-MEMS-based photoacoustic microscopy system for tracking and visualization of the balloon catheter. M, mirror; c, collimator; OL, objective lens, CL, correction lens, TR, transducer; BC, beam combiner; AL, acoustic lens; I, iris; MEMS, Micro Electro Mechanical Systems.

Figure 1a shows the balloon catheter and the inflation pump used in this study. Figure 1b shows an enlarged balloon catheter tip. A 2.0 × 20-mm balloon dilatation catheter (Medtronic, Sprinter Legend^®^) for coronary balloon angioplasty was used. The balloon catheter consists of a balloon with an optically transparent polyether block amide and a core made by polyvinyl chloride (PVC) marked with platinum. The balloon was inflated to a pressure of 6 atm using an inflation device.

### 2.2. Fast Photoacoustic Microscopic Imaging

As shown in Figure 1c, the f-MEMS-PAM system was used to visualize the balloon catheter. In order to generate PA wave with a tiny beam size, the nano-second pulsed laser beam was emitted from a diode laser (SPOT-10-200-532, Elforlight, Daventry, UK) with a center wavelength of 532 nm, a pulsed width of 6 ns, and a repetition rate of 10 kHz. To achieve in vivo imaging, we used the laser energy around 10 mJ/cm^2^ (Supplemental Material), which satisfied the ANSI limits (Max 20 mJ/cm^2^ at visible light). The laser beam was then reshaped by an iris (SM1D12, Thorlabs, Newton, NJ, USA) which was held by a right-angle kinematic mount (KCB1E, Thorlabs, Newton, NJ, USA) that worked as a spatial filter. Two mirrors were inclined at a 45-degree angle of vertical and horizontal planes to adjust the direction of the beam to the collimator (F280APC-A, Thorlabs) before inserting into a single-mode optical fiber (P1-405BPM-FC-1, Thorlabs). The diverged beam from the optical fiber became the collimated beam after passing through the second collimator (F260APC-A, Thorlabs). The collimated beam was again focused by an objective lens (AC254-060-A, Thorlabs). It then penetrated through the beam combiner and projected into the sample. The beam combiner in front of the unfocused ultrasound transducer constituted by a normal prism and aluminum-coated prism could confocal align the laser beam with the ultrasound focus. A plano-concave lens (NT45-010, Edmund, Tucson, AZ, USA) as an acoustic lens was utilized to focus the acoustic beam used to support acoustic focusing on the transducer. Volumetric scanning was conducted with a 1-axis MEMS scanner (OpitchoMS-001, Opticho Inc., Ltd., Pohang, Korea) and a linear stepper motor stage (L-509-10SD00, PI) which achieved 25 Hz B-scan imaging controlled by a data acquisition (DAQ) board (PCIe-6321, NI instruments, Austin, TX, USA). The PA signal went back to the beam combiner. It was immediately detected by a high-frequency ultrasonic transducer (V214-BC-RM, 50 MHz, Olympus, Tokyo, JPN) and an RF-amplifier (ZX60-3018G-S+, Mini-Circuit, Brooklyn, NY, USA). The high-speed digitizer (ATS9371, AlazarTech Pointe-Claire, QC, Canada) digitalized PA signals with 12 bit and 1 GS/s sampling rates from the transducer. The whole system was controlled by a LabView program (NI instruments, Austin, TX, USA). Our system can achieve high-resolution imaging with 12 µm for lateral resolution and 45 µm for axial resolution [48]. Single B-scan and volumetric 3D PAM images were acquired at 0.25 and 12 s, respectively, which is a relatively fast imaging acquisition rate compared to other group reports [33,47,48,49]. Maximum projection amplitude (MAP) and cross-sectional PAM images were acquired and analyzed with MATLAB (R2016a, Mathworks, Natick, MA, USA). Furthermore, volumetric 3D PAM images and movies were reconstructed and produced with Amira program (Amira 6, FEI).

### 2.3. Automatic Surface Removing Algorithm

**Figure 2:**
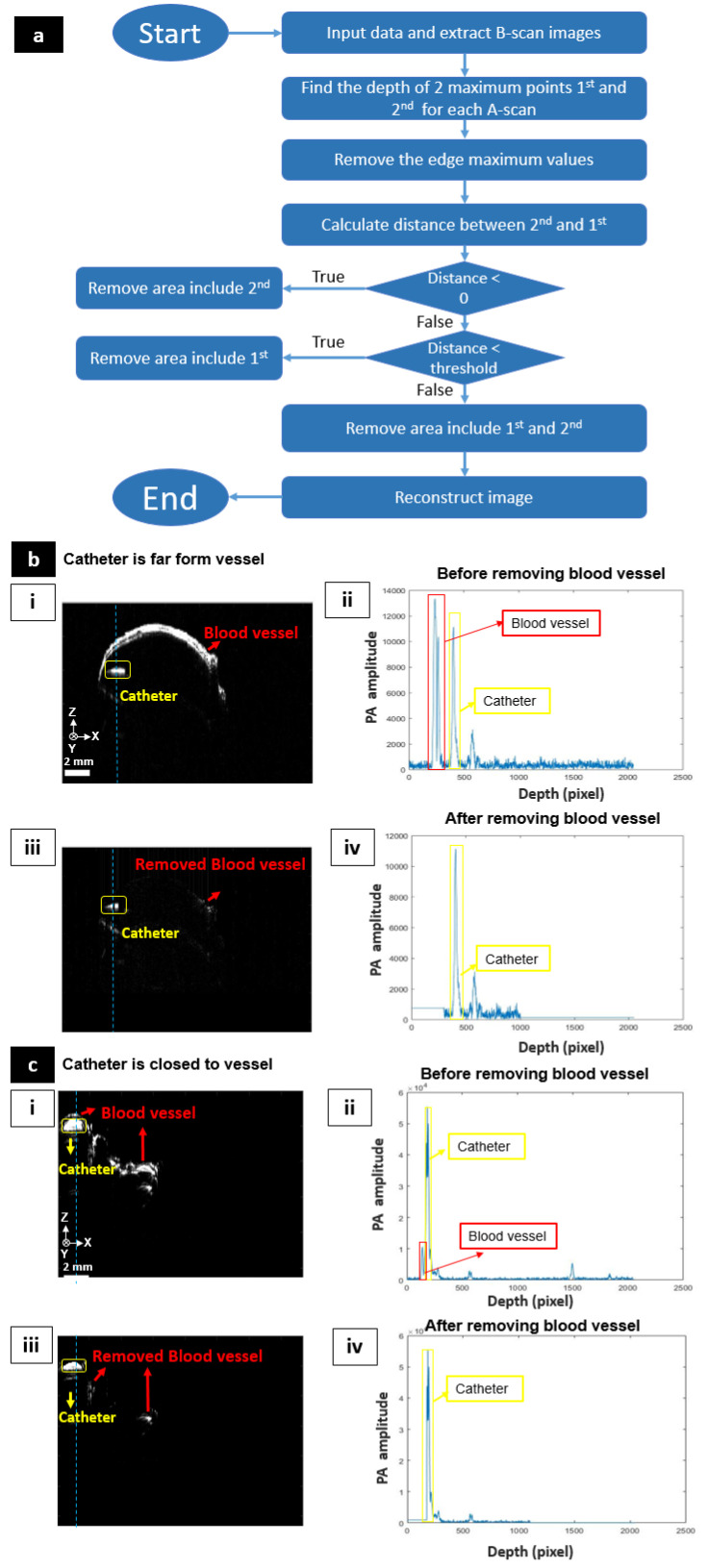
Surface removing process. (**a**) Flow chart showing the automatic surface removing algorithm. (**b**,**c**) Before/after applying the surface removing process when the catheter is far from the vessel and close to the vessel, respectively. (**i**,**ii**) Cross-sectional photoacoustic (PA) image and the selected depth-resolved A-scan profile before adapting the surface removing process, respectively. (**iii**,**iv**) Cross-sectional PA image and depth-resolved A-scan profile after adapting the surface removing process, respectively.

Due to the strong light absorption of blood at a wavelength of 532 nm, PAI can show blood vessel networks without any contrast agent [50]. Unfortunately, in the current systemic condition, the catheter was fully surrounded by blood vessels. These blood vessels usually generate the biggest signals. As a result, the MAP PAM image only shows the image of blood vessels. For delineating the correct location of the balloon catheter in blood vessels, these unexpected PA signals from blood vessels should be removed. Normally, the largest signals that appear on B-scan images are those for upper walls of blood vessels and catheters, in which the signal for blood vessels is larger. When the catheter is quite close to the walls of blood vessels, these signals will overlap. If the catheter’s signal is too small, signals of upper and lower walls of the blood vessel are the two largest signals. Therefore, it is critical to identify these two signals from each A-scan for removing unwanted blood vessel signals. In order to distinguish between the upper blood vessel signal and the catheter signal, the depth of the signal should be considered. As shown in Figure 2b, the signal of the catheter is always deeper in all cases. Thus, in the two largest signals, the signal with a greater depth will be the catheter. In the case of the two largest signals belonging to blood vessels, a distance threshold is needed. The threshold should be smaller than the width of the blood vessel. If the distance between the two signals is less than the threshold, the second signal will belong to the catheter; otherwise, it will belong to the lower blood vessel wall. In this study, we used a threshold of 300 pixels. Random noise also should be considered. Noise may become the largest signal and appear on the MAP image. Interfering signals are often concentrated on the upper or lower edge of the MAP image. They can be eliminated by removing all large signals at the top and bottom edges of the image. The algorithm is summarized in Figure 2a. Figure 2b(i,ii) show the B-scan image and A-scan profile before applying the removing process, respectively. The biggest signal and the second biggest signal belong to blood vessel and catheter, respectively. The blood vessel signal is almost removed after adapting the surface removing process, as shown in Figure 2b(iii). In Figure 2b(iv), the signal of the blood vessel area becomes the constant line, and the biggest signal belongs to the catheter. Even if there is another signal next to the catheter signal, it will not affect the image reconstruction process because only the largest signal is selected, and the MAP image will only show the signal of the catheter. In this case, the signal from the surface and the catheter core has a sufficient distance. So, the removal process works well. Unfortunately, as shown in Figure 2c(i,ii), some regions show that the catheter is located very close to the surface of the blood vessel. In this case, there is trouble in removing the surface successfully. Although we enable to extract the only core region, we should consider the loss of the PA signals from the core as shown in Figure 2c(iii,iv).

### 2.4. Animal Preparing

A 12-week-old male Sprague-Dawley rat weighing 380 g (Samtako, Korea) was used after one week of acclimation. The study protocol was approved by the Institutional Animal Ethical Committee of Chonnam National University Hospital. A rat was anesthetized with an intraperitoneal injection of Ketamine (80 mg/kg)/Xylazine (12 mg/kg). Skin and soft tissue incisions were made from the xiphoid process to both the femoral region. After exposing the abdominal aorta with around 1.1 mm diameter and both iliac arteries, proximal ligation at the highest abdominal aorta was obtained using a 4–0 silk suture. Distal ligations of both common iliac arteries approximately 1 cm from the iliac bifurcation were prepared in the same manner. Arteriotomy was performed through the upper half of the vessel circumference with surgical scissors at a location of 1–2 mm close to the distal ligation site. After removing arterial blood using gauzes, a guidewire was inserted through the arteriotomy and advanced to the proximal ligation site. The balloon catheter was then passed over a guidewire into the abdominal aorta. Beginning at the distal ligation part of the common iliac artery, the balloon was totally deflated. When the balloon was positioned at the abdominal aorta, PAM images were acquired in three medium conditions in the balloon: no air, air and water. After sacrificing the rat with overdose of the anesthetic mixture, we cut out of the aorta, and the inside of the aorta was washed three or more times with normal saline to confirm that there was no blood or thrombus in the blood vessels. After that, we conducted the same process of in vivo experiment with PAM.

## 3. Results

### 3.1. PA signal Characteristics of The Balloon Catheter

**Figure 3:**
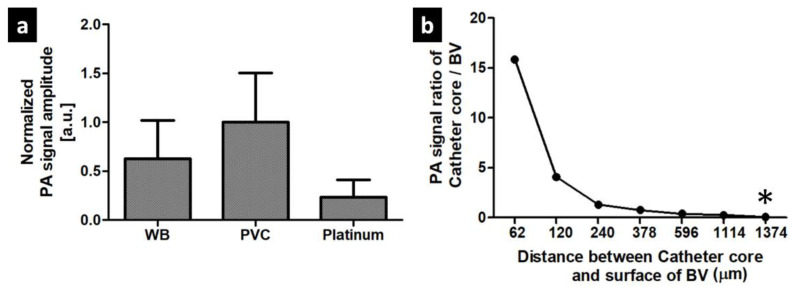
PA characteristics of the balloon catheter. (**a**) PA signals comparison among whole blood (WB), polyvinyl chloride (PVC), and platinum (**b**) PA signal ratio of the catheter core versus the blood vessel (BV) surface at the different distance between the catheter core and the BV surface. * mark indicates the invisible catheter core.

As shown in Figure 3a, PA signals of whole blood and the catheter core components, such as PVC and platinum, were measured under the same PAM setup at 532 nm wavelength. The used laser energy is approximately 3 mJ/cm^2^. Compared to whole blood and platinum, PVC exhibited approximately 38% and 83% higher PA signals at the 532 nm wavelength. Thus, even though the use of 532 nm wavelength laser disturbed the detection of the catheter core directly because of the high PA signal from the blood vessel wall, we estimated that the catheter core also could be detectable due to the relatively high absorption of PVC. Figure 3b shows the PA signal ratio of the catheter core versus the blood vessel surface at the different distance between the catheter core and the surface of the blood vessel. We conducted ex vivo PA signal test with the extracted blood vessel and the inserted balloon catheter. By injecting water slowly into the balloon, we changed the distance between the catheter core and the blood vessel surface. The used laser energy is approximately 10 mJ/cm^2^. As shown in Figure 3b, the maximum visible depth is about 1110 µm with a very low signal of the catheter core. The * mark indicates the invisible depth of the catheter core which is around 1370 µm. By increasing the distance between the catheter core and the blood vessel surface, the PA signal ratio was decreased exponentially. 

### 3.2. The Balloon Catheter Visualization

**Figure 4:**
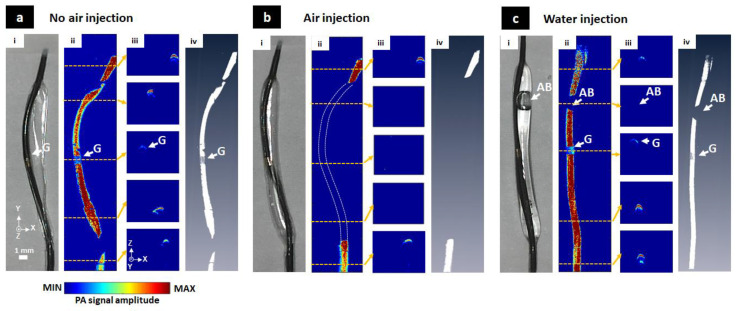
Maximum amplitude projection (MAP), cross-sectional and volumetric 3-dimentional photoacoustic microscopy (3D PAM) images of the balloon catheter with different injection conditions. (**a**) No air injection condition, (**b**) Air injection condition, (**c**) Water injection condition, (**i**) Photographs of the balloon catheter, (**ii**) Corresponding MAP PAM images, (**iii**) Five selected cross-sectional PAM images, (**iv**) Corresponding volumetric 3D PAM images (Movie 1, Movie 2, Movie 3). AB, air bubble; G, gold.

As shown in Figure 4, MAP, cross-sectional and volumetric 3D PAM images of the catheter were obtained with different injection medium conditions for the balloon catheter (i.e., no air injection, air injection and water injection). When there was no air inside, the image of the catheter core was displayed as shown in Figure 4a(ii–iv) because of its black color. There were two locations (two junction points between the core of the catheter and the balloon due to air remaining) without signal on the PAM MAP, causing a loss of PA signal for the image. Air was then injected into the catheter as shown in Figure 4b(i). In Figure 4b(ii–iv), the entire PA signal of the catheter in the air area disappeared due to a strong loss of acoustic attenuation in air. The only non-balloon area was visualized. In Figure 4c(i), water was pumped into the balloon while still keeping a small air bubble (AB) inside the catheter. The core of the catheter was displayed in the water but completely lost in the air bubble area (Figure 4c(ii–iv)). The gold area (G) in the middle of the catheter core was also visualized in Figure 4a(ii,iii), Figure 4c(ii,iii) with relatively low PA signal level. The PA signal of the catheter core showed 231.96% higher than that in the G region for no air injection case and 248.25% for the water injection case. All these results were also shown in volumetric 3D images (Figure 4a(iv), Figure 4b(iv), and Figure 4b(iv)) and movies (Movie 1, Movie 2, and Movie 3). This simple experiment was performed to pre-check the ability of PAM to visualize the catheter at different injection medium conditions before conducting in-vivo and ex-vivo experiments. These obtained results demonstrated the feasibility of using the PAM system to monitor the balloon catheter with high-resolution in real-time.

### 3.3. In Vivo Visualization of the Balloon Catheter

**Figure 5:**
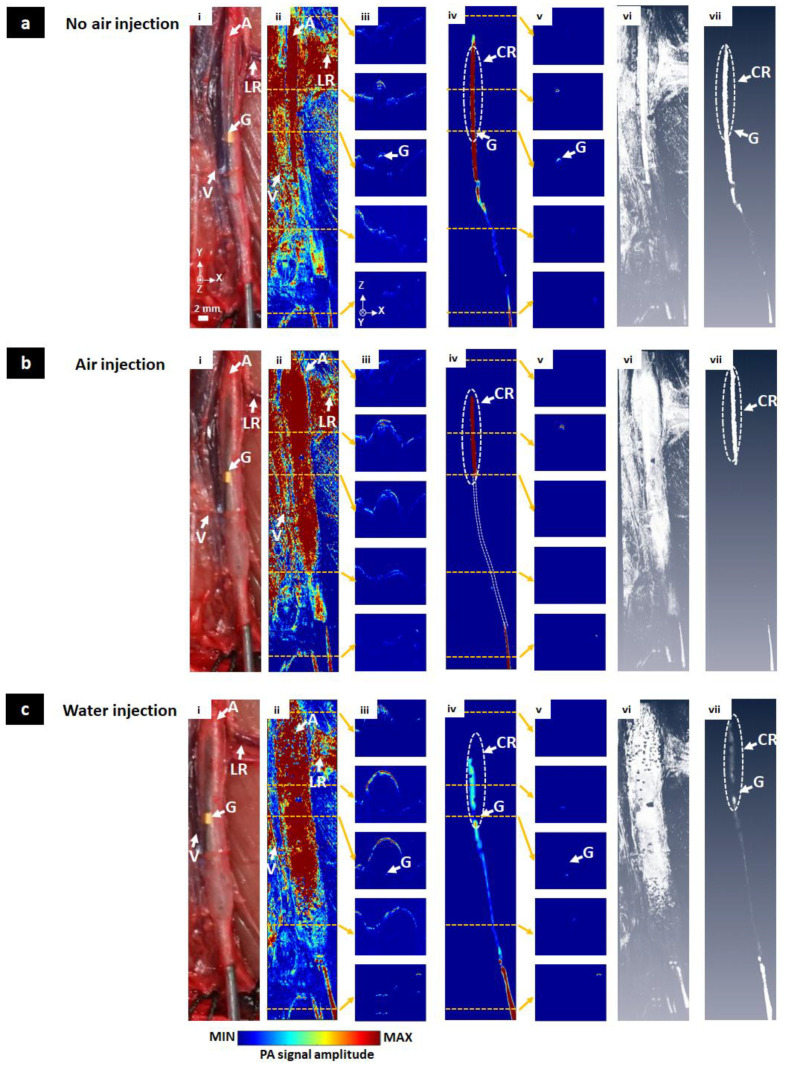
In vivo MAP, cross-sectional, volumetric 3D PAM images of the balloon catheter with different injection conditions. (**a**) No air injection condition, (**b**) Air injection condition, (**c**) Water injection condition, (**i**) Photographs of the balloon catheter, (**ii**) MAP PAM images without the surface removing process, (**iii**) Five selected cross-sectional PAM images without the surface removing process, (**iv**) MAP PAM images with the surface removing process, (**v**) Five selected cross-sectional PAM images with the surface removing process, (**vi**,**vii**) Volumetric 3D PAM images before and after the surface removing process (Movie 4, Movie 5, Movie 6). G, gold; CR, contacted region; A, aorta; LR, left renal artery; V, vein.

The catheter was inserted into the aorta of an anesthetized rat carefully. The results are shown in Figure 5. Under no air condition (Figure 5a), the whole blood vessel area was shown in the MAP PAM image (Figure 5a(ii)) without the surface removing process. In cross-sectional PAM images (Figure 5a(iii)), even the wall of the blood vessel showed the highest PA signal. Because the aorta receives blood through the small-sized microvessel network (called as vasa vasorum), the wall of blood vessel normally generates high PA signals regardless of the internal state of the aorta. The core of the catheter was also observed with a relatively low PA signal level. After applying the surface removing process, the core of the catheter was observed as shown in Figure 5a(iv). These results also were confirmed by volumetric 3D PAM images and a 3D rendering movie (Figure 5a(vi,vii), Movie 4). The signal marked with an oval white dot line (CR, contact region) was the most prominent part because it was adjacent to the blood vessel wall. Due to the actions of arterial muscles that changed the position of the inner catheter, this was easily identified when compared to the balloon catheter in Figure 4a. The lower part of the catheter core far away from the blood vessel wall was significantly reduced in the signal which was less 57.80% than the corresponding part of the blood vessel. The G region maintained the PA signal level because the core of the catheter was very close to the blood surface. Under air injection condition, the MAP image and corresponding cross-sectional images are shown in Figure 5b(ii,iii), respectively. In Figure 5b(iv), after removing the blood vessel, the signal again appeared in the CR area and the non-balloon area. The rest covered by the air medium did not show any signal of the catheter core. Only the background was shown. The same results also were confirmed in 3D rendering data (Figure 5b(vi,vii), Movie 5). Figure 5b(iv,v) show the MAP image and cross-sectional images under water injection condition, respectively. Water was the ideal medium for acoustic signal propagation. Thus, the signal of the catheter was fully collected in the case of water injection not only before removing the surface (Figure 5c(ii,iii)), but also after blood vessel removing (Figure 5c(iv,v)). We also observe the same results from volumetric 3D data (Figure 5c(vi,vii), Movie 6). The pumping of water, however, increased the distance of the scanner with the catheter. Thus, the received signal was quite weak compared to the biggest signals of no air injection case. The PA signal of the catheter core was less 78.66% than blood surface signals. The G region even had a signal 71.24% (no air injection) and 33.62% (water injection) lower than the black color of the core, although it could be easily visualized under no air condition and water injection. 

### 3.4. Ex Vivo Visualization of the Balloon Catheter

**Figure 6:**
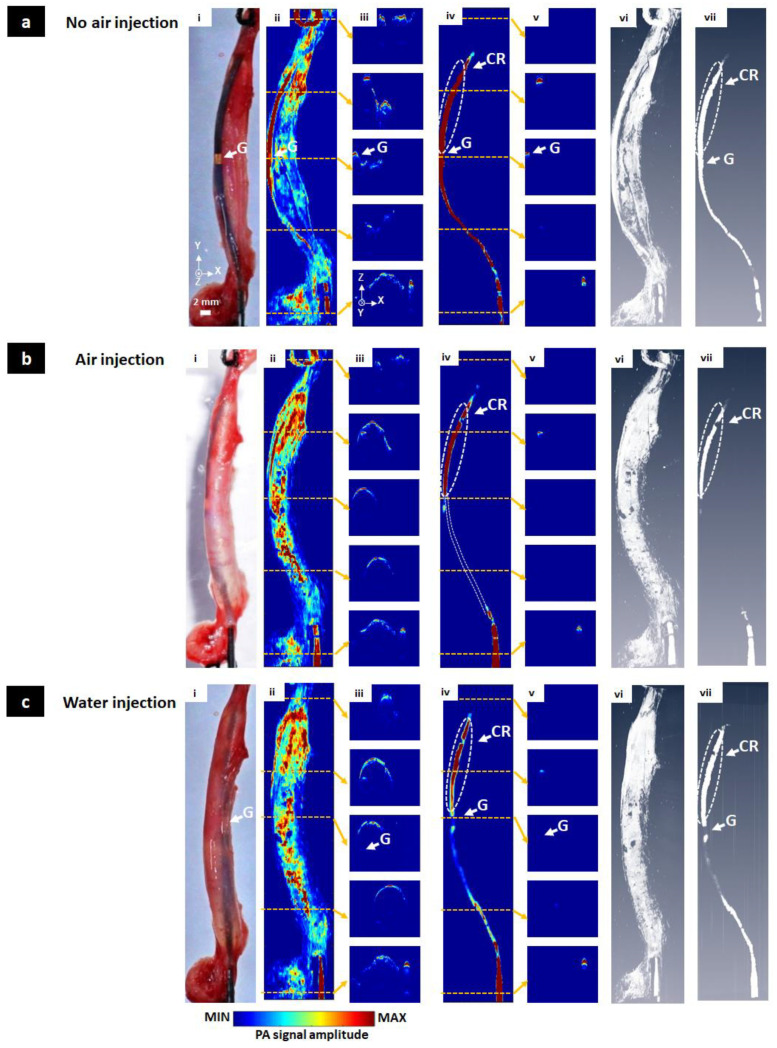
Ex vivo MAP, cross-sectional, and volumetric 3D PAM images of the balloon catheter with different injection conditions. (**a**) No air injection condition, (**b**) Air injection condition, (**c**) Water injection condition, (**i**) Photographs of the balloon catheter, (**ii**) MAP PAM images without the surface removing process, (**iii**) Five selected cross-sectional PAM images without the surface removing process, (**iv**) MAP PAM images with the surface removing process, (**v**) Five selected cross-sectional PAM images with the surface removing process, (**vi**,**vii**) Volumetric 3D PAM images before and after the surface removing process (Movie 7, Movie 8, Movie 9). G, gold; CR, contacted region.

The aorta with the inner core catheter was cleverly separated from the body of the mouse. The position of the balloon catheter (Figure 6a(i)) was similar to that shown in Figure 4a. In Figure 6a(ii), the core catheter signal was outstanding compared to the remaining parts. After removing the surface, the catheter was fully shown in Figure 6a(iv). All cross-sectional images (Figure 6a(v)) included the signal. The PA signal level is almost maintained due to the short distance between the blood vessel and the catheter. Volumetric 3D data also showed the same results (Figure 6a(vi,vii), Movie 7). Results under air injection conditions, including MAP, cross-sectional, and volumetric 3D images before and after the surface removing process were shown in Figure 6b(ii–vii, Movie 8). Similar to the in vivo case, only the nearest surface area of the catheter core indicated by the CR area was detected regardless of medium condition. PA signal of another region was not detected. Finally, water injection was conducted as shown in Figure 6c(i). In Figure 6c(ii), the blood vessel signal after water injection overwhelmed the remaining signals, causing the signal of the core to be greatly reduced. However, the whole catheter including the G area could be monitored and visualized as shown in Figure 6c(iv). The PA signal of the catheter core was 60.64% less than blood surface signals. These results were also observed in Figure 6c(vi,vii) and Movie 9.

## 4. Discussion

We demonstrated the feasibility of tracking and visualizing of the balloon catheter using the PAM technique under open surgery condition. Our approach successfully provided the precise tracking location of the catheter, as well as the inserted catheter shape in an unknown blood vessel. By providing high-resolution of cross-sectional and MAP PAM images, we could estimate whether the catheter was placed well in the aorta and whether the function of the balloon was working normally or not. Furthermore, at different injecting medium conditions in the balloon, we could acquire the black colored core of the catheter selectively without any PA contrast agent in ex/in vivo experiments. First, under no air injection condition, PA signals and the shape of the catheter core were almost shown in PAM images. Second, under air injection condition, all PA signals in the balloon disappeared because acoustic loss caused high acoustic attenuation in air. Third, under water injection condition, whole PA signals were detected again. Thus, we could successfully generate PAM images. Although direct contacted regions between the catheter core and the blood vessel wall showed high PA signals regardless of inserted medium conditions because of the intrinsic bending shape of the catheter, other contactless areas of the balloon catheter successfully worked despite uncontrollable in vivo experiments.

Unfortunately, although we carried out the tracking of the balloon catheter with PAM as the proof of concept, it still needs to improve its systemic performance for overcoming current limitations and further clinical study. First, the only use of a 532 nm pulsed laser beam with high blood absorption required an additional surface removing process that caused unexpected signal loss and post-processing time. As shown in/ex vivo MAP PAM images in Figure 4 and Figure 5, there was a lot of signal loss due to unavoidable contracts between the catheter core and the blood vessel. Eventually, this lost information at the front end of the catheter interfered with correct catheter guidance. To overcome this issue, near-infrared region light (NIR, i.e., 680 nm–1800 nm) source and PA contrast agents such as MB and ICG can be solutions [51,52]. If NIR light can be used, more light energy will reach the catheter due to low light absorption and scattering in total hemoglobin [53,54]. NIR light provides an opportunity to avoid high PA signals in vascular and deep PA imaging. Furthermore, the balloon catheter filled with PA contrast agents will enhance the sensitivity of PA images. By choosing a specific wavelength laser such as 680 nm for MB and 800 nm for ICG, we can expect selective PA imaging of the balloon catheter.

Second, the current PAM imaging setup is not proper for clinical translation. Even though PAM provides high spatial resolution, its intrinsic imaging configuration which combination of the focused laser and acoustic beam makes PAM weak in visualizing deep tissue imaging. As shown in Figure 3b, in our experimental approach, we only enabled to show approximately 1 mm in a shallow depth. Therefore, for clinical implementation in the minimally invasive surgery such as hybrid operation, the handheld probe or laparoscopic probe should be updated [55]. To utilize PAI in the general catheter guiding, that works in several centimeter depths, a clinically based PAI system should be applied [56]. Furthermore, combining PA contrast agents and selecting the appropriate excitation light wavelength can be better identification of the balloon catheter in deep tissue. Additionally, the use of clinically approved PA agents is a better option than water in patient safety during the catheter guiding.

## 5. Conclusions

In this study, we conducted the tracking and visualization of the balloon catheter with PAM. Despite the limited single wavelength laser at 532 nm, we successfully demonstrated the potential of PAI technology for tracking the balloon catheter by applying the simple surface removing process and fast and high-resolution PAM scanning. These results were verified on in/ex vivo animal experiments. As a next plan, we will guide the balloon catheter in medium and large animals using clinical PAI systems, multi-wavelength lasers and PA contrast agents. We believe that the results of this study and advanced next trials will improve the possibility of clinical translation in the near future.

## Supplementary Materials

Supplementary materials can be found online at: https://www.mdpi.com/1424-8220/20/19/5585/s1.
