# Supplementary material for: Feasibility Study of Precise Balloon Catheter Tracking and Visualization with Fast Photoacoustic Microscopy
Source: Sensors (Basel). 2020 Sep 29;20(19):5585. doi: 10.3390/s20195585 (PMC7582572; doi:10.3390/s20195585)
Supplement: Supplementary file 1 [file sensors-20-05585-s001.zip › sensors-911878-author resubmit -supplementary/Supplementary Material-sensors.docx]

**Supplementary Materials**

Manuscript tile: Feasibility study of Precise balloon catheter tracking and visualization with fast photoacoustic microscopy

Authors: Jahae Kim, Thi Thao Mai, Jin Young Kim, Jung-Joon Min, Chulhong Kim, and

Changho Lee*

1. **Calculation of the used laser energy on the sample**

Frist, we calculated the beam width (W(z)) on the vessel by the following steps.

1. Suppose that the used PAM laser beam has the Gaussian beam shape (Figure S1); (2) Based on $Z_{R}=\pi{w_{o}}^{2}$/λ , we extract the Rayleigh distance (Z_R_); (3) From $w\left( z \right)=w_{o}\sqrt{1+{(Z/Z_{R})}^{2}}$, the Gaussian beam (w(z)) width can be estimated. $w_{o} , \lambda, Z$note that half of the spot size, the used laser wavelength, and the imaging depth.


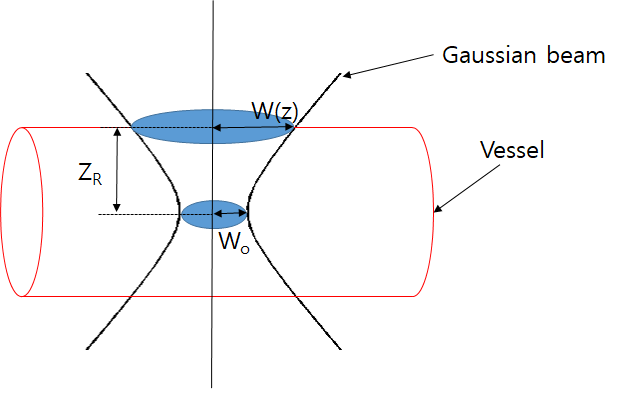


Figure S2. The schematic of the Gaussian beam in the vessel.

Second, we calculated the use of energy (mJ/cm^2^) using the estimated beam area on the vessel and the measured laser power. Measured laser power and repetition rates are 4 mW and 10 kHz.

Finally, the estimated laser energy is calculated as 10.07 mJ/cm^2^.
