# Supplementary figures and images for: Feasibility Study of Precise Balloon Catheter Tracking and Visualization with Fast Photoacoustic Microscopy
Source: Sensors (Basel). 2020 Sep 29;20(19):5585. doi: 10.3390/s20195585 (PMC7582572; doi:10.3390/s20195585)

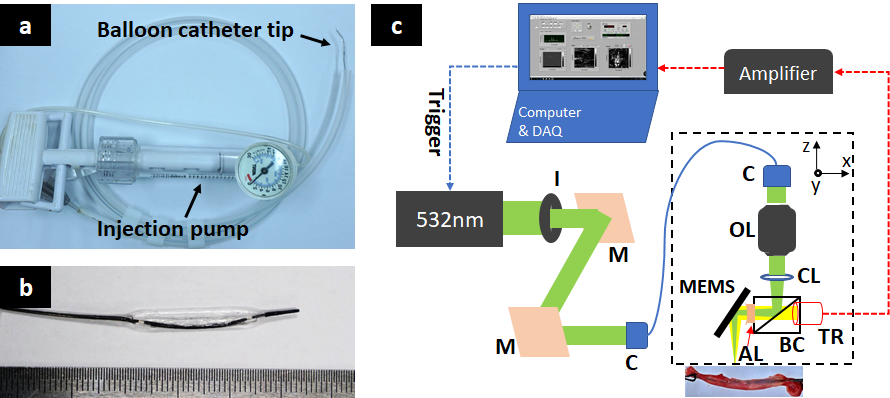

Supplement: Supplementary file 1 [file sensors-20-05585-s001.zip › sensors-911878-author resubmit -supplementary/sensors-911878-author resubmit -figures/figure1.tif]

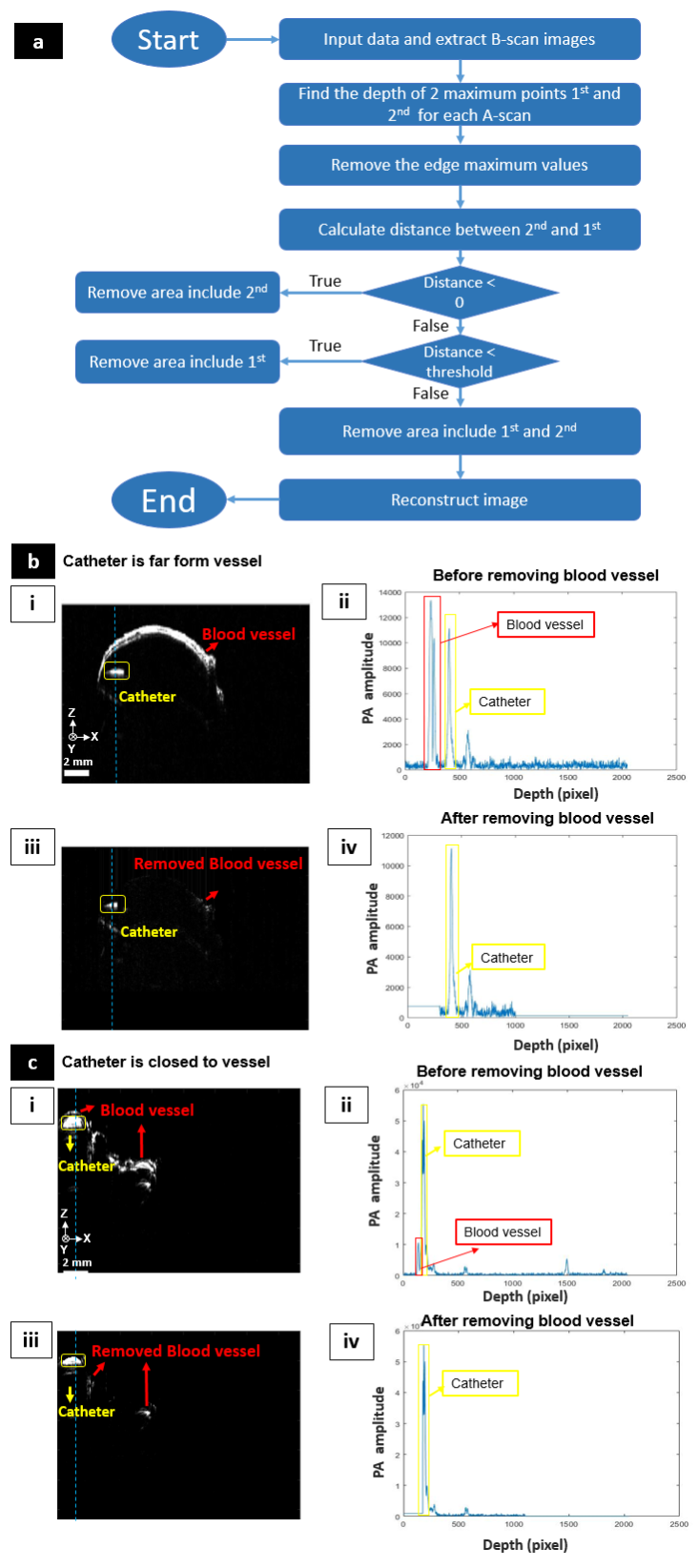

Supplement: Supplementary file 1 [file sensors-20-05585-s001.zip › sensors-911878-author resubmit -supplementary/sensors-911878-author resubmit -figures/figure2.tif]

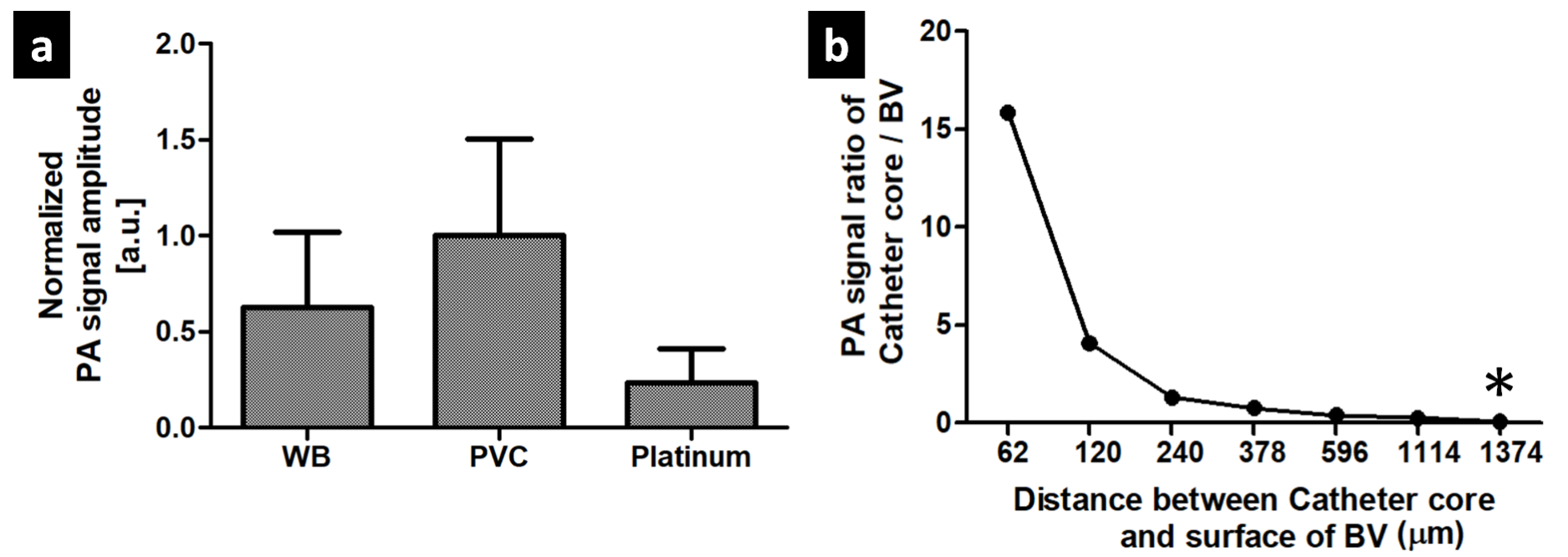

Supplement: Supplementary file 1 [file sensors-20-05585-s001.zip › sensors-911878-author resubmit -supplementary/sensors-911878-author resubmit -figures/figure3.tif]

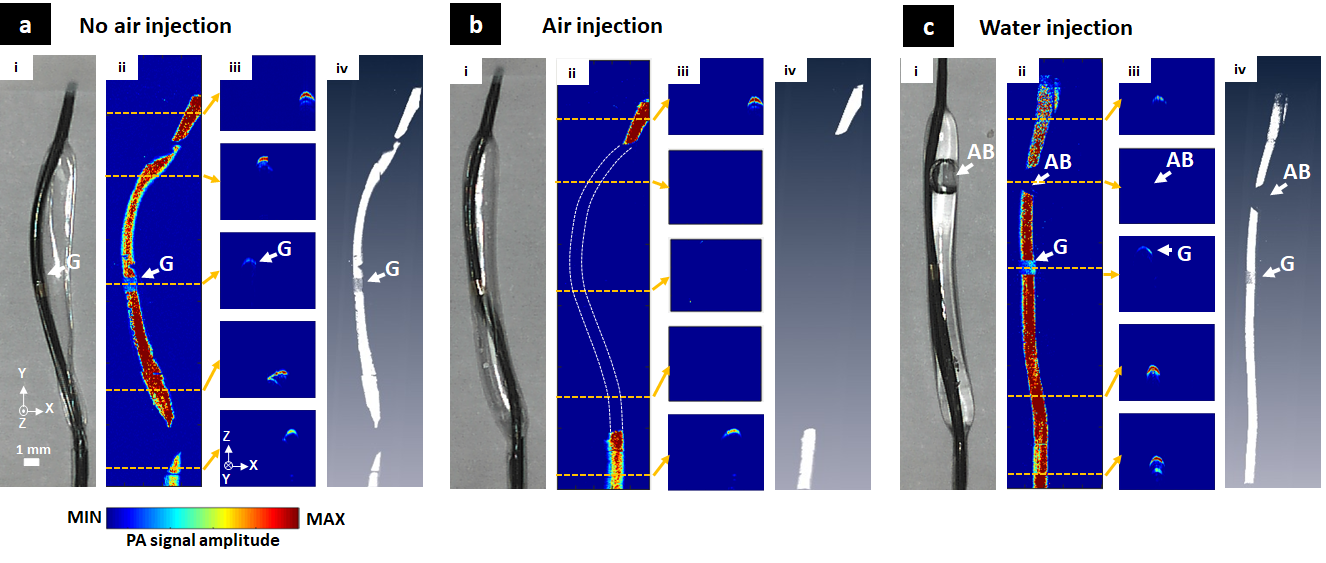

Supplement: Supplementary file 1 [file sensors-20-05585-s001.zip › sensors-911878-author resubmit -supplementary/sensors-911878-author resubmit -figures/figure4.tif]

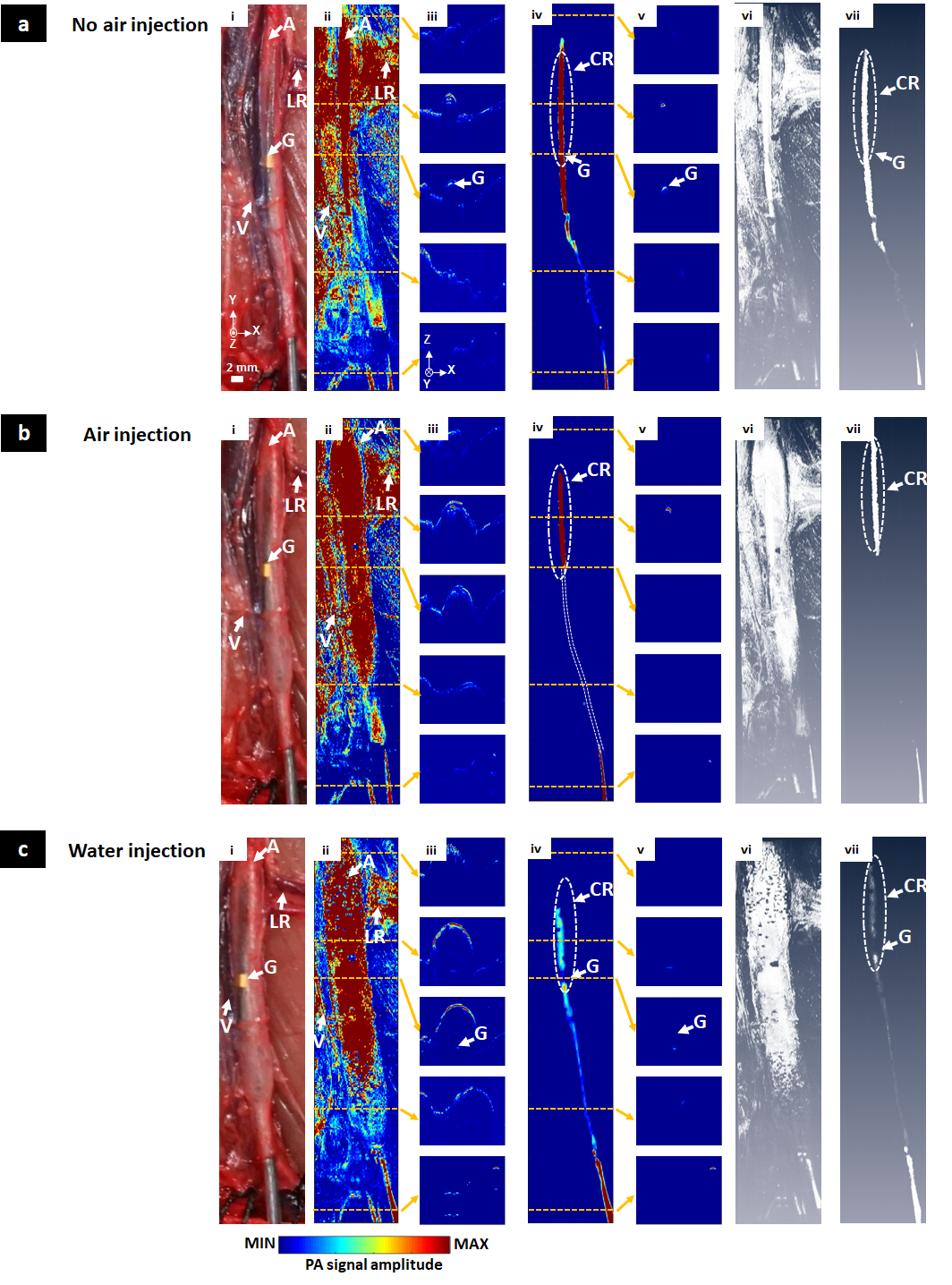

Supplement: Supplementary file 1 [file sensors-20-05585-s001.zip › sensors-911878-author resubmit -supplementary/sensors-911878-author resubmit -figures/figure5.tiff]

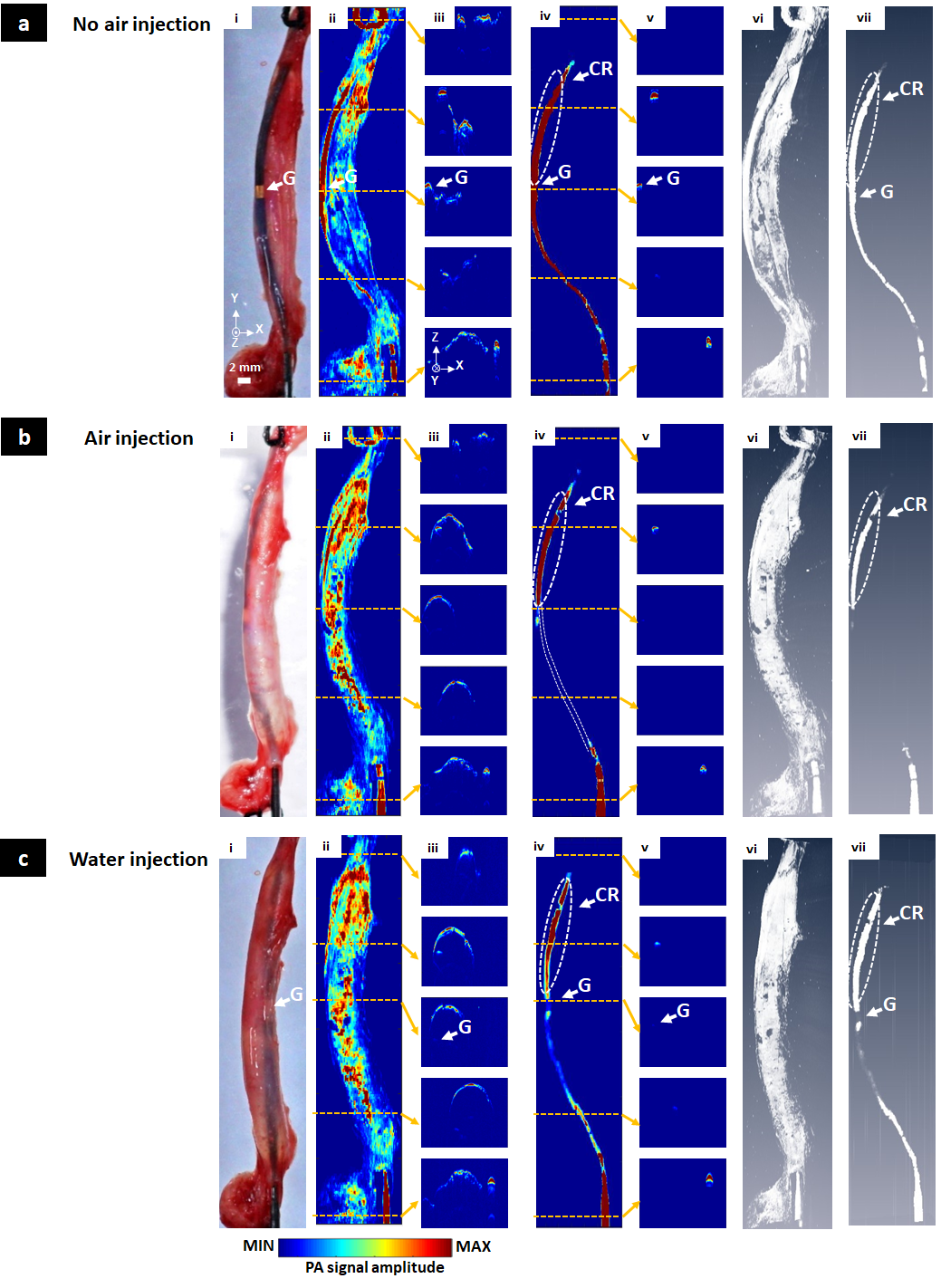

Supplement: Supplementary file 1 [file sensors-20-05585-s001.zip › sensors-911878-author resubmit -supplementary/sensors-911878-author resubmit -figures/figure6.tif]
